# Biased Adjusted Poisson Ridge Estimators-Method and Application

**DOI:** 10.1007/s40995-020-00974-5

**Published:** 2020-10-03

**Authors:** Muhammad Qasim, Kristofer Månsson, Muhammad Amin, B. M. Golam Kibria, Pär Sjölander

**Affiliations:** 1 Department of Economics, Finance and Statistics, Jönköping University, Jönköping, Sweden; 2 Department of Statistics, University of Sargodha, Sargodha, Pakistan; 3 Department of Mathematics and Statistics, Florida International University, Miami, FL USA

**Keywords:** Maximum likelihood estimator, Multicollinearity, Poisson ridge regression, Modified almost unbiased ridge estimators, Mean square error

## Abstract

Månsson and Shukur (Econ Model 28:1475–1481, 2011) proposed a Poisson ridge regression estimator (PRRE) to reduce the negative effects of multicollinearity. However, a weakness of the PRRE is its relatively large bias. Therefore, as a remedy, Türkan and Özel (J Appl Stat 43:1892–1905, 2016) examined the performance of almost unbiased ridge estimators for the Poisson regression model. These estimators will not only reduce the consequences of multicollinearity but also decrease the bias of PRRE and thus perform more efficiently. The aim of this paper is twofold. Firstly, to derive the mean square error properties of the Modified Almost Unbiased PRRE (MAUPRRE) and Almost Unbiased PRRE (AUPRRE) and then propose new ridge estimators for MAUPRRE and AUPRRE. Secondly, to compare the performance of the MAUPRRE with the AUPRRE, PRRE and maximum likelihood estimator. Using both simulation study and real-world dataset from the Swedish football league, it is evidenced that one of the proposed, MAUPRRE ($\hat{k}_{q4}$) performed better than the rest in the presence of high to strong (0.80–0.99) multicollinearity situation.

**Electronic supplementary material:**

The online version of this article (10.1007/s40995-020-00974-5) contains supplementary material, which is available to authorized users.

## Introduction

The Poisson regression model (PRM) is a special form of the generalized linear models and is used when the dependent variable is collected in terms of counts of nonnegative integers. A PRM adopts a Poisson distribution for the dependent variable and assumes the log of its expected value can be modeled by a linear combination of relevant parameters. The model is commonly applied for counts such as the occurrence rate of an event (counts) per unit of time. These counts must be independent to facilitate that one count will not make another event to be more or less likely. Instead, the probability of a count per unit of time is related to independent variables such as, e.g., the time of day. Examples of likely Poisson processes could be the number of infected patients per day at a clinic, a country’s number of bankruptcies per year, the number of vehicles per hour passing through a freeway toll. The maximum likelihood estimator (MLE) is used to estimate the unknown regression coefficients of the PRM. This estimator is considered to be the best estimator for the PRM, and as long as under- or overdispersion is not present in the data set, this is a standard model for these types of count problems. However, in the presence of multicollinearity problems, the mean square error (MSE) of the MLE become unstable with high variances of the regression coefficients and the inference based on MLE may not be reliable. Another consequence of multicollinearity is the wider confidence intervals, decreased statistical power which result in increased probabilities of type II errors in the parameters’ hypothesis tests. In addition, the uncertainty of the estimated coefficients is higher because of an increased coefficient variance due to multicollinearity.

Many biased estimation techniques have been proposed for linear regression models to reduce multicollinearity, such as the ridge regression estimator by Hoerl and Kennard (1970) and the Liu estimator by Liu (1993). Moreover, Nomura (1988) developed an almost unbiased ridge estimator in the linear regression model, thus with the cost of a very low bias, but substantially more efficient as compared to the ordinary ridge regression under certain conditions. Månsson and Shukur (2011) proposed a Poisson ridge regression estimator (PRRE) to reduce the effects of problems associated with multicollinear data. Kibria et al. (2015) proposed a number of biasing parameters, and Asar and Genç (2018) suggested a two-parameter biased estimator in the PRM to adjust for multicollinearity. Türkan and Özel (2016) developed Almost Unbiased PRRE (AUPRRE) and Modified AUPPRE (MAUPRRE). Kaçıranlar and Dawoud (2018) examined the performance of Poisson and negative binomial ridge predictors. Algamal and Alanaz (2018) proposed different methods to estimate the value of ridge parameter (*k*) for PRRE. Rashad and Algamal (2019) proposed a new ridge regression approach in the PRM to reduce the issue of collinearity between explanatory variables, and recently Qasim et al. (2019) proposed a Liu-type of regression estimator for the PRM. Türkan and Özel (2016) did not discuss the MSE properties of AUPRRE and MAUPRRE and not derive the optimal value of the ridge parameter (*k*). However, no published research work seems available regarding the MSE properties of the AUPRRE and MAUPRRE and their optimal ridge estimators for the PRM.

The main contribution of this paper is twofold. One is to derive the MSE properties of the MAUPRRE and AUPRRE. Second is, by simulations and by the empirical application in terms of MSE and bias, to compare the performance of the MAUPRRE with the AUPRRE, PRRE and MLE. In addition, we introduce new estimating methods for estimate the value of ridge parameter (*k*) for AUPRRE and MAUPRRE and the performance of proposed ridge estimators is compared with the existing estimators by considering different factors in the simulation study. Furthermore, the intuitive, and empirical relevance of the MAUPRRE and AUPRRE are exhibited by employing an estimation of a real-world dataset, where we systematically investigate which estimator that to the highest degree can remedy the effects of multicollinearity. In this empirical application, we model the number of goals scored at away games (as a function of the quality of the teams measured by bookmaker odds). By this approach, it is easily demonstrated that the standard errors and the estimated MSEs of proposed estimators are decreased substantially as compared to the existing estimators in the presence of multicollinearity problem. Hence, the precision of the estimated parameters is increased, which of course is one of the main objectives of demonstrating the method in an empirical situation.

The rest of the article is organized as follows: in Sect. 2, we define the model of interest and MLE, PRRE, AUPRRE and MAUPRRE. The MSE properties are derived in Sect. 3. In Sect. 4, the optimal value of the ridge parameter is derived, and we propose new ridge estimators for estimating the value of ridge parameter, *k* for AUPRRE and MAUPPRE. Monte Carlo simulation and its results are presented in Sect. 5. In Sect. 6, the advantages of our proposed ridge estimators are illustrated by using our estimators to analyze an empirical dataset based on the Swedish football league. Finally, the concluding remarks of article are discussed in Sect. 7.

## Methodology

This section illustrates the model of interest and characteristic of different estimators.

### The Poisson Regression Model

The PRM is applicable only when the dependent variable deals with count data. Suppose $y_{i}$ is the dependent variable and follows a Poisson distribution with parameter $(\mu_{i} )$ and it can be denoted as ${\rm P}\left( {\mu_{i} } \right)$ with probability mass function

1 $$ f\left( {y_{i} } \right) = \frac{{e^{{\mu_{i} }} \mu_{i}^{{y_{i} }} }}{{y_{i} !}},\quad y_{i} = 0,1,2, \ldots  \quad i = 1,2, \ldots ,n . $$

The PRM is commonly developed by using the canonical link function, such that $\mu_{i} = \exp \left( {x_{i}^{t} \beta } \right)$, where $x_{i}$ is the *i*th row of $X$ which is an $n \times \left( q \right)$ data matrix with $q$ non-stochastic explanatory variables, $\beta$ is a $q \times 1$ vector of the unknown regression coefficients. The log-likelihood function is defined as

2 $$ \begin{aligned} l = l\left( {\mu ;y} \right) & = \mathop \sum \limits_{i = 1}^{n} \left\{ {y_{i} \ln \left( {\mu_{i} } \right) - \mu_{i} - \ln \left( {\mathop \prod \limits_{i = 1}^{n} y_{i} !} \right)} \right\} \\ & = \mathop \sum \limits_{i = 1}^{n} \left\{ {y_{i} \left( {x_{i}^{t} \beta } \right) - \exp \left( {x_{i}^{t} \beta } \right) - \ln \left( {\mathop \prod \limits_{i = 1}^{n} y_{i} !} \right)} \right\}. \\ \end{aligned} $$

The traditional MLE is used to estimate the unknown regression coefficients for the PRM. The MLE is obtained by taking the first order derivative of Eq. (2) with respect to $\beta$:

3 $$ S\left( \beta \right) = \frac{\partial l}{\partial \beta } = \mathop \sum \limits_{i = 1}^{n} \left( {y_{i} - \exp \left( {x_{i}^{t} \beta } \right)} \right)x_{i} = 0, $$

where $S\left( \beta \right)$ is the score function, since Eq. (3) is nonlinear in $\beta$, we estimate the unknown coefficients through iterative weighted least squares. Let $\beta^{\left( m \right)}$ be the estimated value of MLE of $\beta$ with *m* iterations which may be written as

4 $$ \beta^{{\left( {m + 1} \right)}} = \beta^{\left( m \right)} + \left\{ {I\left( {\beta^{\left( m \right)} } \right)} \right\}^{ - 1}  S\left( {\beta^{\left( m \right)} } \right), $$

where $I\left( {\beta^{\left( m \right)} } \right) = \left\{ { - E\left( {\frac{{\partial^{2} L}}{{\partial \beta \partial \beta^{t} }}} \right)} \right\}$ is a $q \times q$ Fisher information matrix and both $S\left( {\beta^{\left( m \right)} } \right)$ and $I\left( {\beta^{\left( m \right)} } \right)$ are evaluated at $\beta^{\left( m \right)}$. At convergence in deviance of Eq. (4), the MLE is found by applying the following iterative weighted least squares method

5 $$ \hat{\beta }_{\text{MLE}} = \left( {X^{t} \hat{W}X} \right)^{ - 1} X^{t} \hat{W}{\text{z}}^{*} , $$

where $\hat{W} = {\text{diag}} \left\{ {\hat{\mu }_{1} ,\hat{\mu }_{2} , \ldots ,\hat{\mu }_{n} } \right\},\;z^{*} = \log \left( {\hat{\mu }_{i} } \right) + \frac{{y_{i} - \hat{\mu }_{i} }}{{\hat{\mu }_{i} }}$, is the adjusted response variable. Both $\hat{W}$ and ${\text{z}}^{*}$ are evaluated by Fisher’s scoring iterative procedure (see, e.g., Hardin et al. 2007).

In order to obtain the MSEs of the parameters, we consider $\varLambda = {\text{diag}}\left( {\lambda_{1} ,\lambda_{2} , \ldots ,\lambda_{q} } \right) = {\mathbb{Q}}^{t} \left( {X^{t} \hat{W}X} \right){\mathbb{Q}} = {\mathcal{Z}}^{t} \hat{W}{\mathcal{Z}}$, where ${\mathcal{Z}} = X{\mathbb{Q}},\;{\mathbb{Q}}$ is the orthogonal matrix whose columns are the eigenvectors of $X^{t} \hat{W}X$ and $\lambda_{1} \ge \lambda_{2} \ge , \ldots , \ge \lambda_{q} > 0$ are the eigenvalues of the matrix $X^{t} \hat{W}X$, respectively. The $\hat{\beta }_{\text{MLE}}$ can be written as

$$ \begin{aligned} \tilde{\gamma }_{\text{MLE}} & = \left( \varLambda \right)^{ - 1} {\mathcal{Z}}^{t} \hat{W}z^{*} , \\ \tilde{\beta }_{\text{MLE}} & = {\mathbb{Q}}\tilde{\gamma }_{\text{MLE}} . \\ \end{aligned} $$

The covariance matrix of the $\tilde{\beta }_{\text{MLE}}$ is defined as

6 $$ {\text{Cov}}\left( {\tilde{\beta }_{\text{MLE}} } \right) = \left( \varLambda \right)^{ - 1} . $$

In addition, the scalar MSE of the $\tilde{\beta }_{\text{MLE}}$ is defined as

7 $$ {\text{MSE}}\left( {\tilde{\beta }_{\text{MLE}} } \right) = E\left( {\tilde{\beta }_{\text{MLE}} - \beta } \right)^{t} \left( {\tilde{\beta }_{\text{MLE}} - \beta } \right) = {\text{tr}}\left\{ {\varLambda^{ - 1} } \right\} = \mathop \sum \limits_{j = 1}^{q} \frac{1}{{\lambda_{j} }}, $$

where $\lambda_{j}$ is the *j*th eigenvalue of the ${\mathcal{Z}}^{t} \hat{W}{\mathcal{Z}}$ matrix.

### The Poisson Ridge Regression Estimator

It can be easily seen that the MSE of the MLE becomes overstated when the explanatory variables are linearly correlated because some of the eigenvalues will be small and ${\mathcal{Z}}^{t} \hat{W}{\mathcal{Z}}$ is ill-conditioned. To reduce the effects of multicollinearity, Månsson and Shukur (2011) proposed a PRRE estimator which can be defined as

$$ \hat{\beta }_{\text{PRRE}} = \left( {X^{t} \hat{W}X + kI_{q} } \right)^{ - 1} X^{t} \hat{W}X\hat{\beta }_{\text{MLE}} $$

The $\hat{\beta }_{\text{PRRE}}$ can be written as

8 $$ \tilde{\beta }_{\text{PRRE}} = \left( {\varLambda_{{kI_{q} }} } \right)^{ - 1} {\mathcal{Z}}^{t} \hat{W}z^{*} , $$

where $\varLambda_{{kI_{q} }} = {\text{diag}}\left( {\lambda_{1} + kI_{q} ,\lambda_{2} + kI_{q} , \ldots ,\lambda_{q} + kI_{q} } \right)$ and *k* ($k > 0$) is the ridge parameter. The bias, covariance matrix and MSE of the $\tilde{\beta }_{\text{PRRE}}$ are, respectively, defined as

$$ {\text{Bias}}\left( {\tilde{\beta }_{\text{PRRE}} } \right) = E\left( {\tilde{\beta }_{\text{PRRE}} } \right) - \beta $$

9 $$ {\text{Bias}}\left( {\tilde{\beta }_{\text{PRRE}} } \right) = - k\varLambda^{ - 1}_{{kI_{q} }} \beta , $$

10 $$ {\text{Cov}}\left( {\tilde{\beta }_{\text{PRRE}} } \right) = \varLambda^{ - 1}_{{kI_{q} }} \varLambda \varLambda^{ - 1}_{{kI_{q} }} , $$

11 $$ {\text{MSE}}\left( {\tilde{\beta }_{\text{PRRE}} } \right) = \varLambda^{ - 1}_{{kI_{q} }} \varLambda \varLambda^{ - 1}_{{kI_{q} }} + k^{2} \varLambda^{ - 1}_{{kI_{q} }} \beta \beta^{t} \varLambda^{ - 1}_{{kI_{q} }} , $$

where $\varLambda_{{kI_{q} }} = {\text{diag}}\left( {\lambda_{1} + kI_{q} ,\lambda_{2} + kI_{q} , \ldots ,\lambda_{q} + kI_{q} } \right)$ and $\varLambda = {\text{diag}}\left( {\lambda_{1} ,\lambda_{2} , \ldots ,\lambda_{q} } \right) = {\mathcal{Z}}^{t} \hat{W}{\mathcal{Z}}$, where ${\mathbb{Q}}$ is the orthogonal matrix whose columns are the eigenvectors of ${\mathcal{Z}}^{t} \hat{W}{\mathcal{Z}}$. The scalar MSE of the PRRE is obtained by applying the tr(.) operator on Eq. (11), which can be defined as

12 $$ {\text{MSE}}\left( {\tilde{\beta }_{\text{PRRE}} } \right) = \mathop \sum \limits_{j = 1}^{q} \left( {\frac{{\lambda_{j} }}{{\left( {\lambda_{j} + k} \right)^{2} }}} \right) + \mathop \sum \limits_{j = 1}^{q} \left( {\frac{{k^{2} \alpha_{i}^{2} }}{{\left( {\lambda_{j} + k} \right)^{2} }}} \right) = \mathop \sum \limits_{j = 1}^{q} \left( {\frac{{\lambda_{j} + k^{2} \alpha_{i}^{2} }}{{\left( {\lambda_{j} + k} \right)^{2} }}} \right), $$

where $\alpha = \varUpsilon^{t} \hat{\beta }_{\text{MLE}}$, $\gamma$ is the eigenvector of the matrix ${\mathcal{Z}}^{t} \hat{W}{\mathcal{Z}}$ and *k* is the ridge parameter of the PRRE.

### Almost Unbiased Poisson Ridge Regression Estimator

The PRRE overcome the problem of multicollinearity, but this estimator has a large bias. Therefore, Türkan and Özel (2016) proposed AUPRRE. This estimator cannot only remedy the problem of multicollinearity but also reduce the bias as compared to PRRE and MLE. Before explaining the full AUPRRE, we first define the almost unbiased ridge estimator in Definition 2.3.1:

#### Definition 2.3.1

Xu and Yang (2011), Consider $\hat{\beta }$ is a biased estimator of the parameter $\beta$ and the bias vector $\hat{\beta }$ is given by ${\text{Bias}}\left( {\hat{\beta }} \right) = E\left( {\hat{\beta }} \right) - \beta = {\rm M}\beta$, which shows that $E\left( {\hat{\beta } - {\rm M}\beta } \right) = \beta$, then the estimator $\tilde{\beta } = \hat{\beta } - {\rm M}\beta = \left( {I - {\rm M}} \right)\hat{\beta }$ is called the almost unbiased estimator based on the biased estimator $\hat{\beta }$.

Below, we define the AUPRRE based on the PRRE. According to Definition 2.3.1, we define the following AUPRRE based on ${\text{Bias}}\left( {\hat{\beta }_{\text{PRRE}} } \right) = \left( {X^{t} \hat{W}X + kI_{q} } \right)^{ - 1} X^{t} \hat{W}X\hat{\beta }_{\text{MLE}} - \beta$:

$$ \begin{aligned} \hat{\beta }_{\text{AUPRRE}} & = \left[ {I - \left\{ {\left( {X^{t} \hat{W}X + kI_{q} } \right)^{ - 1} X^{t} \hat{W}X - I} \right\}} \right]\hat{\beta }_{\text{PRRE}} \\ & = \left[ {2I - \left( {X^{t} \hat{W}X + kI_{q} } \right)^{ - 1} X^{t} \hat{W}X} \right]\hat{\beta }_{\text{PRRE}} \\ & = \left[ {I + \left( {X^{t} \hat{W}X + kI_{q} } \right)^{ - 1} X^{t} \hat{W}X} \right]\left( {X^{t} \hat{W}X + kI_{q} } \right)^{ - 1} X^{t} \hat{W}X\hat{\beta }_{\text{MLE}} \\ & = \left[ {I + k\left( {X^{t} \hat{W}X + kI_{q} } \right)^{ - 1} } \right]\left[ {I - k\left( {X^{t} \hat{W}X + kI_{q} } \right)^{ - 1} } \right]\hat{\beta }_{\text{MLE}} \\ & = \left[ {I - \left\{ {k\left( {X^{t} \hat{W}X + kI_{q} } \right)^{ - 1} } \right\}^{2} } \right]\hat{\beta }_{\text{MLE}} . \\ \end{aligned} $$

The above expression can be defined as

13 $$ \tilde{\beta }_{\text{AUPRRE}} = \left[ {I - \left\{ {k\left( {\varLambda_{{kI_{q} }} } \right)^{ - 1} } \right\}^{2} } \right]\tilde{\gamma }_{\text{MLE}} . $$

The bias, covariance matrix and MSE of the $\tilde{\beta }_{\text{AUPRRE}}$ are defined, respectively, as following:

$$ {\text{Bias}}\left( {\tilde{\beta }_{\text{AUPRRE}} } \right) = E\left( {\tilde{\beta }_{\text{AUPRRE}} } \right) - \beta $$

14 $$ {\text{Bias}}\left( {\tilde{\beta }_{\text{AUPRRE}} } \right) = - k^{2} \varLambda^{ - 2}_{{kI_{q} }} \beta , $$

15 $$ {\text{Cov}}\left( {\tilde{\beta }_{\text{AUPRRE}} } \right) = \left( {I_{q} - k^{2} \varLambda^{ - 2}_{{kI_{q} }} } \right)\varLambda^{ - 1} \left( {I_{q} - k^{2} \varLambda^{ - 2}_{{kI_{q} }} } \right). $$

16 $$ {\text{MSE}}\left( {\tilde{\beta }_{\text{AUPRRE}} } \right) = \left( {I_{q} - k^{2} \varLambda^{ - 2}_{{kI_{q} }} } \right)\varLambda^{ - 1} \left( {I_{q} - k^{2} \varLambda^{ - 2}_{{kI_{q} }} } \right) + k^{4} \varLambda^{ - 2}_{{kI_{q} }} \beta \beta^{t} \varLambda^{ - 2}_{{kI_{q} }} . $$

The scalar MSE of the AUPRRE is obtained by applying the tr(.) operator on Eq. (16), which can be stated as

17 $$ {\text{MSE}}\left( {\tilde{\beta }_{\text{AUPRRE}} } \right) = \mathop \sum \limits_{j = 1}^{q} \frac{1}{{\lambda_{j} }}\left( {1 - \frac{{k^{2} }}{{\left( {\lambda_{j} + k} \right)^{2} }}} \right)^{2} + \mathop \sum \limits_{j = 1}^{q} \left( {\frac{{k^{4} \alpha_{i}^{2} }}{{\left( {\lambda_{j} + k} \right)^{4} }}} \right). $$

### Modified Almost Unbiased Poisson Ridge Regression Estimator

Türkan and Özel (2016) proposed a modified Jackknifed ridge estimator or MAUPRRE for the PRM by following the work of Singh et al. (1986). The MAUPRRE is defined as

$$ \hat{\beta }_{\text{MAUPRRE}} = \left[ {I_{q} - \left\{ {k\left( {X^{t} \hat{W}X + kI_{q} } \right)^{ - 1} } \right\}^{2} } \right]\left[ {I_{q} - \left\{ {k\left( {X^{t} \hat{W}X + kI_{q} } \right)^{ - 1} } \right\}} \right]\hat{\beta }_{\text{MLE}} . $$

The $\hat{\beta }_{\text{MAUPRRE}}$ can be written as

18 $$ \tilde{\beta }_{\text{MAUPRRE}} = \left[ {I_{q} - \left\{ {k\left( {\varLambda_{{kI_{q} }} } \right)^{ - 1} } \right\}^{2} } \right]\left[ {I_{q} - \left\{ {k\left( {\varLambda_{{kI_{q} }} } \right)^{ - 1} } \right\}} \right]\tilde{\gamma }_{\text{MLE}} $$

The bias, variance, MMSE and scalar MSE of the $\tilde{\beta }_{\text{MAUPRRE}}$ are defined as

$$ {\text{Bias}}\left( {\tilde{\beta }_{\text{MAUPRRE}} } \right) = E\left( {\hat{\beta }_{\text{MAUPRRE}} } \right) - \beta $$

19 $$ {\text{Bias}}\left( {\tilde{\beta }_{\text{MAUPRRE}} } \right) = k\varLambda^{ - 2}_{{kI_{q} }} \left( {I_{q} + k\varLambda^{ - 1}_{{kI_{q} }} - k^{2} \varLambda^{ - 2}_{{kI_{q} }} } \right)\beta , $$

20 $$ {\text{Cov}}\left( {\tilde{\beta }_{\text{MAUPRRE}} } \right) = \left( {I_{q} - k^{2} \varLambda^{ - 2}_{{kI_{q} }} } \right)\left( {I_{q} - k\varLambda^{ - 1}_{{kI_{q} }} } \right)\varLambda^{ - 1} \left( {I_{q} - k\varLambda^{ - 1}_{{kI_{q} }} } \right)\left( {I_{q} - k^{2} \varLambda^{ - 2}_{{kI_{q} }} } \right). $$

21 $$ {\text{MSE}}\left( {\tilde{\beta }_{\text{MAUPRRE}} } \right) = {\text{Cov}}\left( {\tilde{\beta }_{\text{MAUPRRE}} } \right) + {\text{Bias}}\left( {\tilde{\beta }_{\text{MAUPRRE}} } \right){\text{Bias}}\left( {\tilde{\beta }_{\text{MAUPRRE}} } \right)^{t} . $$

The scalar MSE of the ${\text{MAUPRRE}}$ is obtained by applying the tr(.) operator on Eq. (21), which can be stated as

22 $$ {\text{MSE}}\left( {\tilde{\beta }_{\text{MAUPRRE}} } \right) = \mathop \sum \limits_{j = 1}^{q} \left\{ {\frac{1}{{\lambda_{j} }}\left( {1 - \frac{{k^{2} }}{{\left( {\lambda_{j} + k} \right)^{2} }}} \right)^{2} \left( {\frac{{\lambda_{j} }}{{\left( {\lambda_{j} + k} \right)^{2} }}} \right)^{2} } \right\} + \mathop \sum \limits_{j = 1}^{q} \left( {\frac{{k^{2} \alpha_{i}^{2} }}{{\left( {\lambda_{j} + k} \right)^{2} }}} \right)\left( {1 + \frac{k}{{\left( {\lambda_{j} + k} \right)^{2} }} - \frac{{k^{2} }}{{\left( {\lambda_{j} + k} \right)^{2} }}} \right) $$

## Mean Square Error Properties of the Estimators

In this section, we derive the MSE properties of the AUPRRE and MAUPRRE for the PRM. We also make a comparison of the AUPRRE and MAUPRRE with the existing estimators such as MLE and PRRE. We show the superiority of the AUPRRE and MAUPRRE under different conditions. The performance of $\tilde{\beta }_{\text{MLE}}$, $\tilde{\beta }_{\text{PRRE}}$, $\tilde{\beta }_{\text{AUPRRE}}$ and $\tilde{\beta }_{\text{MAUPRRE}}$ is theoretically judged by using MSE and the bias criteria. Therefore, we define Lemma 3.1 for comparison purpose.

### Lemma 3.1

(Farebrother 1976) Let *M* $\left( {M > 0} \right)$ be a positive definite matrix, $\varTheta$ be a vector of nonzero constants and *c* is a positive constant, then $cM - \alpha \alpha^{t} \ge 0$ if and only if $\alpha^{t} M^{ - 1} \alpha \le c$.

### Comparison of $\tilde{\beta }_{\text{AUPRRE}}$ with $\tilde{\beta }_{\text{PRRE}}$ and $\tilde{\beta }_{\text{MLE}}$

#### Theorem 3.1.1

In the PRM, we have ${\text{Bias}}\left( {\tilde{\beta }_{\text{AUPRRE}} } \right)^{2} < {\text{Bias}}\left( {\tilde{\beta }_{\text{PRRE}} } \right)^{2}$ for $k > 0$.

#### Proof

By using Eqs. (9) and (14), we have

$$ \begin{aligned} \Delta_{1} & = {\text{Bias}}\left( {\tilde{\beta }_{\text{PRRE}} } \right)^{2} - {\text{Bias}}\left( {\tilde{\beta }_{\text{AUPRRE}} } \right)^{2} \\ & = k^{2} \varLambda_{{kI_{q} }}^{ - 1} \beta \beta^{t} \varLambda_{{kI_{q} }}^{ - 1} - k^{4} \varLambda_{{kI_{q} }}^{ - 2} \beta \beta^{t} \varLambda_{{kI_{q} }}^{ - 2} = \left( {\beta F\beta^{t} } \right), \\ \end{aligned} $$

where $$ F = k^{2} \varLambda_{{kI_{q} }}^{ - 2} - k^{4} \varLambda_{{kI_{q} }}^{ - 4} = {\text{diag}}\left\{ {\frac{{k^{2} \left( {\lambda_{j} + 2k} \right)\lambda_{j} }}{{\left( {\lambda_{j} + k} \right)^{4} }}} \right\}_{j = 1}^{q} > 0, $$ thus for $$ k > 0, $$ the proof is completed.

#### Theorem 3.1.2

If $k > \left( {3 - \lambda_{j} \alpha_{j}^{2} + \sqrt {\left( {3 + \lambda_{j} \alpha_{j}^{2} } \right)^{2} + 4\lambda_{j} \alpha_{j}^{2} } } \right)/4\alpha_{j}^{2}  \quad {\text{for}}\;j = 1,2, \ldots ,q,$, then the $\tilde{\beta }_{\text{AUPRRE}}$ is superior to the $\tilde{\beta }_{PRRE}$ for the PRM in terms of the scalar MSE.

#### Proof

From Eqs. (12) and (17), we have

$$ \begin{aligned} \Delta_{2} & = {\text{MSE}}\left( {\tilde{\beta }_{\text{PRRE}} } \right) - {\text{MSE}}\left( {\tilde{\beta }_{\text{AUPRRE}} } \right) \\ & = \mathop \sum \limits_{j = 1}^{q} \left( {\frac{{\lambda_{j} + k^{2} \alpha_{j}^{2} }}{{\left( {\lambda_{j} + k} \right)^{2} }}} \right) - \mathop \sum \limits_{j = 1}^{q} \frac{1}{{\lambda_{j} }}\left( {1 - \frac{{k^{2} }}{{\left( {\lambda_{j} + k} \right)^{2} }}} \right)^{2} - \mathop \sum \limits_{j = 1}^{q} \left( {\frac{{k^{4} \alpha_{j}^{2} }}{{\left( {\lambda_{j} + k} \right)^{4} }}} \right). \\ & = \mathop \sum \limits_{j = 1}^{q} \left[ {\frac{{\lambda_{j} \left\{ {2\left( {k\alpha_{j} } \right)^{2} + k\lambda_{j} \alpha_{j}^{2} - 3k - 2\lambda_{j} } \right\}k}}{{\left( {\lambda_{j} + k} \right)^{4} }}} \right]. \\ \end{aligned} $$

Since $\Delta_{2}$ is positive definite for $k > 0$ if and only if when $\left\{ {2\left( {k\alpha_{i} } \right)^{2} + k\lambda_{j} \alpha_{i}^{2} - 3k - 2\lambda_{j} } \right\} > 0$ and this expression is a quadratic function of *k* which has following roots

$$ k = {{\left( {3 - \lambda_{j} \alpha_{j}^{2} \pm \sqrt {\left( {3 + \lambda_{j} \alpha_{j}^{2} } \right)^{2} + 4\lambda_{j} \alpha_{j}^{2} } } \right)} \mathord{\left/ {\vphantom {{\left( {3 - \lambda_{j} \alpha_{j}^{2} \pm \sqrt {\left( {3 + \lambda_{j} \alpha_{j}^{2} } \right)^{2} + 4\lambda_{j} \alpha_{j}^{2} } } \right)} {4\alpha_{j}^{2} }}} \right. \kern-0pt} {4\alpha_{j}^{2} }} $$

It is noted that the root ${{\left( {3 - \lambda_{j} \alpha_{j}^{2} - \sqrt {\left( {3 + \lambda_{j} \alpha_{j}^{2} } \right)^{2} + 4\lambda_{j} \alpha_{j}^{2} } } \right)} \mathord{\left/ {\vphantom {{\left( {3 - \lambda_{j} \alpha_{j}^{2} - \sqrt {\left( {3 + \lambda_{j} \alpha_{j}^{2} } \right)^{2} + 4\lambda_{j} \alpha_{j}^{2} } } \right)} {4\alpha_{j}^{2} }}} \right. \kern-0pt} {4\alpha_{j}^{2} }}$ is negative. Thus, if $k > 0$, then ${{k > \left( {3 - \lambda_{j} \alpha_{j}^{2} + \sqrt {\left( {3 + \lambda_{j} \alpha_{j}^{2} } \right)^{2} + 4\lambda_{j} \alpha_{j}^{2} } } \right)} \mathord{\left/ {\vphantom {{k > \left( {3 - \lambda_{j} \alpha_{j}^{2} + \sqrt {\left( {3 + \lambda_{j} \alpha_{j}^{2} } \right)^{2} + 4\lambda_{j} \alpha_{j}^{2} } } \right)} {4\alpha_{j}^{2} }}} \right. \kern-0pt} {4\alpha_{j}^{2} }}$. Thus, the AUPRRE is superior to the PRRE in sense of scalar MSE for the PRM.

#### Theorem 3.1.3

The $\tilde{\beta }_{\text{AUPRRE}}$ is superior to the $\tilde{\beta }_{\text{MLE}}$ in PRM for $k < {{\left( {2\lambda_{j} + \lambda_{j} \sqrt {2\left( {1 + \lambda_{j} \alpha_{j}^{2} } \right)} } \right)} \mathord{\left/ {\vphantom {{\left( {2\lambda_{j} + \lambda_{j} \sqrt {2\left( {1 + \lambda_{j} \alpha_{j}^{2} } \right)} } \right)} {\lambda_{j} \alpha_{j}^{2} - 1 }}} \right. \kern-0pt} {\lambda_{j} \alpha_{j}^{2} - 1 }}$ when $\lambda_{j} \alpha_{j}^{2} - 1 > 0$ and for $k > 0$ when $\lambda_{j} \alpha_{j}^{2} - 1 \le 0$ for $j = 1,2, \ldots ,q.$

#### Proof

From Eqs. (7) and (17), we have

$$ \begin{aligned} \Delta_{3} = & {\text{MSE}}\left( {\tilde{\beta }_{\text{MLE}} } \right) - {\text{MSE}}\left( {\tilde{\beta }_{\text{AUPRRE}} } \right) \\ & = \mathop \sum \limits_{j = 1}^{q} \left( {\frac{1}{{\lambda_{j} }}} \right) - \mathop \sum \limits_{j = 1}^{q} \frac{1}{{\lambda_{j} }}\left( {1 - \frac{{k^{2} }}{{\left( {\lambda_{j} + k} \right)^{2} }}} \right)^{2} - \mathop \sum \limits_{j = 1}^{q} \left( {\frac{{k^{4} \alpha_{j}^{2} }}{{\left( {\lambda_{j} + k} \right)^{4} }}} \right). \\ & = \mathop \sum \limits_{j = 1}^{q} k^{2} \left[ {\frac{{\left( {1 - \lambda_{j} \alpha_{j}^{2} } \right)k^{2} + 4\lambda_{j} k + 2\lambda_{j}^{2} }}{{\lambda_{j} \left( {\lambda_{j} + k} \right)^{4} }}} \right]. \\ \end{aligned} $$

Since $\Delta_{3}$ is positive definite if and only if $\left\{ {\left( {1 - \lambda_{j} \alpha_{j}^{2} } \right)k^{2} + 4\lambda_{j} k + 2\lambda_{j}^{2} } \right\} > 0$ and this expression is a quadratic function of *k.* (i) If $1 > \lambda_{j} \alpha_{j}^{2}$ for $j = 1,2, \ldots ,q,$, then $\left\{ {\left( {1 - \lambda_{j} \alpha_{j}^{2} } \right)k^{2} + 4\lambda_{j} k + 2\lambda_{j}^{2} } \right\} > 0$. (ii) $1 < \lambda_{j} \alpha_{j}^{2}$ for $j = 1,2, \ldots ,q,$ then, we have $k < {{\left( {2\lambda_{j} + \lambda_{j} \sqrt {2\left( {1 + \lambda_{j} \alpha_{j}^{2} } \right)} } \right)} \mathord{\left/ {\vphantom {{\left( {2\lambda_{j} + \lambda_{j} \sqrt {2\left( {1 + \lambda_{j} \alpha_{j}^{2} } \right)} } \right)} {\lambda_{j} \alpha_{j}^{2} - 1}}} \right. \kern-0pt} {\lambda_{j} \alpha_{j}^{2} - 1}}$ and $\left\{ {\left( {1 - \lambda_{j} \alpha_{j}^{2} } \right)k^{2} + 4\lambda_{j} k + 2\lambda_{j}^{2} } \right\} > 0$ by using the method in Theorem 2. Thus, the AUPRRE is superior to the MLE in sense of scalar MSE for the PRM and the proof is completed.

### Comparison of $\tilde{\beta }_{\text{MAUPRRE}}$ with $\tilde{\beta }_{\text{AUPRRE}}$, $\tilde{\beta }_{\text{PRRE}}$ and $\tilde{\beta }_{\text{MLE}}$

#### Theorem 3.2.1

Under the PRM, let $k > 0$ and $b = {\text{Bias}}\left( {\tilde{\beta }_{\text{MAUPRRE}} } \right)$. Then, ${\text{MSE}}\left( {\tilde{\beta }_{\text{MLE}} } \right) - {\text{MSE}}\left( {\tilde{\beta }_{\text{MAUPRRE}} } \right) > 0$ if $b^{t} \left[ {\varLambda^{ - 1} - \mho } \right]^{ - 1} b \le 1$, where $\mho = \left( {I_{q} - k^{2} \varLambda^{ - 2}_{{kI_{q} }} } \right)\left( {I_{q} - k\varLambda^{ - 1}_{{kI_{q} }} } \right)\varLambda^{ - 1} \left( {I_{q} - k\varLambda^{ - 1}_{{kI_{q} }} } \right)\left( {I_{q} - k^{2} \varLambda^{ - 2}_{{kI_{q} }} } \right).$

#### Proof

From Eqs. (6) and (21), the difference between ${\text{MSE}}\left( {\tilde{\beta }_{\text{MLE}} } \right)$ and ${\text{MSE}}\left( {\tilde{\beta }_{\text{MAUPRRE}} } \right)$ is obtained by

$$ \begin{aligned} \Delta_{4} = & {\text{MSE}}\left( {\tilde{\beta }_{\text{MLE}} } \right) - {\text{MSE}}\left( {\tilde{\beta }_{\text{MAUPRRE}} } \right) \\ & = \left[ {\left( {\varLambda^{ - 1} } \right) - \left\{ {\left( {I_{q} - k^{2} \varLambda^{ - 2}_{{kI_{q} }} } \right)\left( {I_{q} - k\varLambda^{ - 1}_{{kI_{q} }} } \right)\varLambda^{ - 1} \left( {I_{q} - k\varLambda^{ - 1}_{{kI_{q} }} } \right)\left( {I_{q} - k^{2} \varLambda^{ - 2}_{{kI_{q} }} } \right)} \right\}} \right] - bb^{t} \\ & = {\text{diag}}\left\{ {\frac{1}{{\lambda_{j} }} - \frac{{\lambda_{j} \left( {\left( {\lambda_{j} + k} \right)^{2} - k^{2} } \right)^{2} }}{{\left( {\lambda_{j} + k} \right)^{6} }}} \right\}_{j = 1}^{q + 1} - bb^{t} \\ \end{aligned} $$

The matrix $\left\{ {\varLambda^{ - 1} - \left( {I_{q} - k^{2} \varLambda^{ - 2}_{{kI_{q} }} } \right)\left( {I_{q} - k\varLambda^{ - 1}_{{kI_{q} }} } \right)\varLambda^{ - 1} \left( {I_{q} - k\varLambda^{ - 1}_{{kI_{q} }} } \right)\left( {I_{q} - k^{2} \varLambda^{ - 2}_{{kI_{q} }} } \right)} \right\}$ is p.d. if $\left( {\lambda_{j} + k} \right)^{6} - \left( {\left( {\lambda_{j} + k} \right)^{2} - k^{2} } \right)^{2} > 0$ where $j = 1,2, \ldots ,q + 1$. Thus, by Lemma 3.1., the proof is completed.

#### Theorem 3.2.2

Under the PRM, let $k > 0$ and $b_{\text{PRRE}} = {\text{Bias}}\left( {\tilde{\beta }_{\text{PRRE}} } \right)$. Then, ${\text{MSE}}\left( {\tilde{\beta }_{\text{PRRE}} } \right) - {\text{MSE}}\left( {\tilde{\beta }_{\text{MAUPRRE}} } \right) > 0$ if $b^{t} \left[ {\varLambda^{ - 1}_{{kI_{q} }} \varLambda \varLambda^{ - 1}_{{kI_{q} }} - \mho } \right]^{ - 1} b \le 1$.

#### Proof

From Eqs. (11) and (21), the difference between ${\text{MSE}}\left( {\tilde{\beta }_{\text{PRRE}} } \right)$ and ${\text{MSE}}\left( {\tilde{\beta }_{\text{MAUPRRE}} } \right)$ is obtained by

$$ \begin{aligned} \Delta_{5} & = {\text{MSE}}\left( {\tilde{\beta }_{\text{PRRE}} } \right) - {\text{MSE}}\left( {\tilde{\beta }_{\text{MAUPRRE}} } \right) \\ & = \left[ {\left\{ {\varLambda^{ - 1}_{{kI_{q} }} \varLambda \varLambda^{ - 1}_{{kI_{q} }} } \right\} - \left\{ {\left( {I_{q} - k^{2} \varLambda^{ - 2}_{{kI_{q} }} } \right)\left( {I_{q} - k\varLambda^{ - 1}_{{kI_{q} }} } \right)\varLambda^{ - 1} \left( {I_{q} - k\varLambda^{ - 1}_{{kI_{q} }} } \right)\left( {I_{q} - k^{2} \varLambda^{ - 2}_{{kI_{q} }} } \right)} \right\}} \right] + b_{\text{PRRE}} b_{\text{PRRE}}^{t} - bb^{t} . \\ & = {\text{diag}}\left\{ {\frac{{\lambda_{j} }}{{\left( {\lambda_{j} + k} \right)^{2} }} - \frac{{\lambda_{j} \left( {\left( {\lambda_{j} + k} \right)^{2} - k^{2} } \right)^{2} }}{{\left( {\lambda_{j} + k} \right)^{6} }}} \right\}_{j = 1}^{q + 1} + b_{\text{PRRE}} b_{\text{PRRE}}^{t} - bb^{t} . \\ \end{aligned} $$

Since $b_{\text{PRRE}} b_{\text{PRRE}}^{t}$ is a nonnegative definite matrix, it is abundant to prove that $\psi = \varLambda^{ - 1}_{{kI_{q} }} \varLambda \varLambda^{ - 1}_{{kI_{q} }} - \mho - bb^{t}$ is p.d. The matrix $\psi$ is p.d. if $\lambda_{j} k^{2} \left( {k^{2} + 4 \lambda_{j} k + 2 \lambda_{j}^{2} } \right)^{2} > 0$, where $j = 1,2, \ldots ,q + 1$. Thus, by Lemma 3.1. The proof is completed.

#### Theorem 3.2.3

Under the PRM, let $k > 0$ and $b_{\text{AUPRRE}} = {\text{Bias}}\left( {\tilde{\beta }_{\text{AUPRRE}} } \right)$ ${\text{MSE}}\left( {\tilde{\beta }_{\text{AUPRRE}} } \right) - {\text{MSE}}\left( {\tilde{\beta }_{\text{MAUPRRE}} } \right) > 0$ if and only if $b^{t} \left[ {\left( {I_{q} - k^{2} \varLambda^{ - 2}_{{kI_{q} }} } \right)\varLambda^{ - 1} \left( {I_{q} - k^{2} \varLambda^{ - 2}_{{kI_{q} }} } \right) - \mho } \right]^{ - 1} b \le 1$.

#### Proof

From Eqs. (11) and (21), the difference between ${\text{MSE}}\left( {\tilde{\beta }_{\text{PRRE}} } \right)$ and ${\text{MSE}}\left( {\tilde{\beta }_{\text{MAUPRRE}} } \right)$ is obtained by

$$ \begin{aligned} \Delta_{6} & = {\text{MSE}}\left( {\tilde{\beta }_{\text{AUPRRE}} } \right) - {\text{MSE}}\left( {\tilde{\beta }_{\text{MAUPRRE}} } \right) \\ & = \left[ {\left\{ {\left( {I_{q} - k^{2} \varLambda^{ - 2}_{{kI_{q} }} } \right)\varLambda^{ - 1} \left( {I_{q} - k^{2} \varLambda^{ - 2}_{{kI_{q} }} } \right)} \right\} - \left\{ {\left( {I_{q} - k^{2} \varLambda^{ - 2}_{{kI_{q} }} } \right)\left( {I_{q} - k\varLambda^{ - 1}_{{kI_{q} }} } \right)\varLambda^{ - 1} \left( {I_{q} - k\varLambda^{ - 1}_{{kI_{q} }} } \right)\left( {I_{q} - k^{2} \varLambda^{ - 2}_{{kI_{q} }} } \right)} \right\}} \right] + b_{\text{AUPRRE}} b_{\text{AUPRRE}}^{t} - bb^{t} . \\ & = {\text{diag}}\left\{ {\frac{1}{{\lambda_{j} }}\left( {1 - \frac{{k^{2} }}{{\left( {\lambda_{j} + k} \right)^{2} }}} \right)^{2} - \frac{{\lambda_{j} \left( {\left( {\lambda_{j} + k} \right)^{2} - k^{2} } \right)^{2} }}{{\left( {\lambda_{j} + k} \right)^{6} }}} \right\}_{j = 1}^{q + 1} + b_{\text{AUPRRE}} b_{\text{AUPRRE}}^{t} - bb^{t} . \\ \end{aligned} $$

Since $b_{\text{AUPRRE}} b_{\text{AUPRRE}}^{t}$ is a nonnegative definite matrix, it is abundant to prove that $\left( {I_{q} - k^{2} \varLambda^{ - 2}_{{kI_{q} }} } \right)\varLambda^{ - 1} \left( {I_{q} - k^{2} \varLambda^{ - 2}_{{kI_{q} }} } \right) - \mho - bb^{t}$ is p.d. $\left[ {\left( {I_{q} - k^{2} \varLambda^{ - 2}_{{kI_{q} }} } \right)\varLambda^{ - 1} \left( {I_{q} - k^{2} \varLambda^{ - 2}_{{kI_{q} }} } \right) - \mho } \right]$ is p.d. if $\lambda_{j} \left( { 2k + \lambda_{j} } \right)^{2} \left( {k^{2} + 2 \lambda_{j} k + \lambda_{j}^{2} - \lambda_{j} } \right) > 0$. Simplifying the last inequality, one can gets $\left( { k + \lambda_{j} } \right)^{2} - \lambda_{j} > 0$, where $j = 1,2, \ldots ,q + 1$. Thus, if $k > 0$, then by Lemma 3.1., the proof is done.

## Proposed Ridge Estimators

It is a complicated challenge for practitioners to select an optimal value of *k*. Therefore, we propose new ridge estimators $\left( {\hat{k}_{q1} - \hat{k}_{q4} } \right)$ for the AUPRRE and MAUPRRE. We also used $\hat{k}_{TO}$ ridge estimator that suggested by Türkan and Özel (2016) for the PRM. Moreover, the performance of $\hat{k}_{q1} - \hat{k}_{q4}$ is compared with the $\hat{k}_{TO}$ in sense of MSE in the simulation and the empirical application sections. In order to obtain an optimal value of the AUPRRE, differentiating the ${\text{MSE}}\left( {\hat{\beta }_{\text{AUPRRE}} } \right)$ with respect to *k* yields Eq. (18):

18 $$ \begin{aligned} \frac{\partial }{\partial k}\left( {MSE_{\text{AUGRRE}} } \right) & = \frac{{ - 4\lambda_{j} k(\lambda_{j} + 2k)}}{{\left( {\lambda_{j} + k} \right)^{5} }} + \frac{{4k^{3} \lambda_{j} \alpha_{j}^{2} }}{{\left( {\lambda_{j} + k} \right)^{5} }} \\ & = \frac{{4\lambda_{j} k\left( {\alpha_{j}^{2} k^{2} - 2k - \lambda_{j} } \right)}}{{\left( {\lambda_{j} + k} \right)^{5} }}. \\ \end{aligned} $$

Let ${{\partial \left\{ {{\text{MSE}}\left( {\hat{\beta }_{\text{AUPRRE}} } \right)} \right\}} \mathord{\left/ {\vphantom {{\partial \left\{ {{\text{MSE}}\left( {\hat{\beta }_{\text{AUPRRE}} } \right)} \right\}} {\partial k}}} \right. \kern-0pt} {\partial k}} = 0$ and resulting function solve for *k,* then we have following optimal value of *k*

19 $$ k_{j} = \frac{{\left\{ {1 + \sqrt {\left( {1 + \alpha_{j}^{2} \lambda_{j} } \right)} } \right\}}}{{\alpha_{j}^{2} }}. $$

Türkan and Özel (2016) concluded that the $k_{TO}$ perform rather well and this estimator is defined as

$$ \hat{k}_{TO} = {\text{median}}\left( {\frac{{\hat{\sigma }^{2} }}{{\hat{\alpha }_{j}^{2} }}} \right), $$

where $\hat{\alpha }_{j}^{2}$ is the *j*th $\left( {j = 1,2, \ldots ,q} \right)$ element of $\varUpsilon^{t} \hat{\beta }_{\text{MLE}}$, $\varUpsilon$ is the eigenvector of matrix $X^{t} \hat{W}X$ and $\hat{\sigma }^{2} = {{\sum\nolimits_{i = 1}^{n} {\left( {y_{i} - \hat{\mu }_{i} } \right)^{2} } } \mathord{\left/ {\vphantom {{\sum\nolimits_{i = 1}^{n} {\left( {y_{i} - \hat{\mu }_{i} } \right)^{2} } } {\left( {n - q + 1} \right)}}} \right. \kern-0pt} {\left( {n - q + 1} \right)}}$. Following ridge estimators are proposed for AUPPRE and MAUPRRE based on the optimal value which derived in Eq. (19).

$$ \begin{array}{*{20}l} {\hat{k}_{q1} = {\text{mean}}\left[ {\frac{{\left\{ {\hat{\sigma }^{2} + \sqrt {\left( {\hat{\sigma }^{2} + \alpha_{j}^{2} \lambda_{j} } \right)} } \right\}}}{{\alpha_{j}^{2} }}} \right].} \hfill & {\hat{k}_{q2} = {\text{median}}\left[ {\frac{{\left\{ {\hat{\sigma }^{2} + \sqrt {\left( {\hat{\sigma }^{2} + \alpha_{j}^{2} \lambda_{j} } \right)} } \right\}}}{{\alpha_{j}^{2} }}} \right].} \hfill \\ {\hat{k}_{q3} = \hbox{max} \left[ {\frac{{\left\{ {\hat{\sigma }^{2} + \sqrt {\left( {\hat{\sigma }^{2} + \alpha_{j}^{2} \lambda_{j} } \right)} } \right\}}}{{\alpha_{j}^{2} }}} \right].} \hfill & {\hat{k}_{q4} = \left[ {\mathop \prod \limits_{j = 1}^{q + 1} \left( {\frac{{\left\{ {\hat{\sigma }^{2} + \sqrt {\left( {\hat{\sigma }^{2} + \alpha_{j}^{2} \lambda_{j} } \right)} } \right\}}}{{\alpha_{j}^{2} }}} \right)} \right]^{{\frac{1}{q + 1}}} .} \hfill \\ \end{array} $$

## The Monte Carlo Simulations

The Monte Carlo simulation study is designed to demonstrate the performance of the estimators. The performance of the proposed estimators is compared with the existing estimators in the sense of MSE and bias under different conditions which are given in Table 1. The dependent variable of the PRM is obtained from the ${\rm P}\left( {\mu_{i} } \right)$ distribution, where

20 $$ \mu_{i} = \exp \left( {\beta_{o} + \beta_{1} x_{i1} + \ldots + \beta_{q} x_{iq} } \right)\quad i = 1,2, \ldots ,n, $$

**Table 1:** The design of the experiment

Name of factors	Notations	Values
Multicollinearity levels	$\rho$	0.80, 0.90, 0.95, 0.99
Number of explanatory variables	$q$	3, 6
Sample sizes	$n$	25, 50, 100, 200, 400
Replications	$R$	5000

We selected the parametric values of $\beta$ under the assumption that $\sum\nolimits_{j = 1}^{q} {\beta_{j}^{2} } = 1$, which are standard restrictions in simulation studies. The correlated explanatory variables are generated as

21 $$ x_{ij} = \sqrt {\left( {1 - \rho^{2} } \right)} z_{ij} + \rho z_{iq + 1} \quad i = 1,2, \ldots , n,\quad j = 1,2, \ldots ,q $$

where $\rho^{2}$ is the correlation between the explanatory variables and $z_{ij}$ represents the independent standard normal pseudo-random numbers. Other factors are also varied in the simulation study such as explanatory variables $\left( {q = 3, 6} \right)$, multicollinearity levels ($\rho = 0.80, 0.90, 0.95, 0.99$) and different sample size. However, the sample sizes need to be increased with the increase in number of explanatory variables to attain the convergence of the iterative weighted least squares algorithm. In order to evaluate the performance of the proposed estimators, the MSE and absolute bias are considered as performance criteria. The MSE and absolute bias are defined as

$$ \begin{aligned} {\text{MSE}}\left( {\hat{\beta }} \right) & = \frac{{\mathop \sum \nolimits_{r = 1}^{R} \left( {\hat{\beta }_{\left( r \right)} - \beta } \right)^{t} \left( {\hat{\beta }_{\left( r \right)} - \beta } \right)}}{R}, \\ {\text{Bias}}\left( {\hat{\beta }} \right) & = \frac{{\mathop \sum \nolimits_{r = 1}^{R} \left| {\hat{\beta }_{\left( r \right)} - \beta } \right|}}{R} \\ \end{aligned} $$

where *R *= 5000 is the total number replications and $\hat{\beta }_{r}$ is the estimate of $\beta$ in the *r*th replication obtained from the MLE, PRRE, AUPRRE and MAUPRRE.

## Results and Discussion

In this subsection, we discuss the simulated MSE and bias of the estimators. The simulated results are shown in Tables 2, 3, 4 and 5. The performance of the estimators is inspected by changing different factors such as the sample size, multicollinearity level and the number of explanatory variables. From Tables 2 and 3, it is clear that the MSE of all the estimators decreases as the same size increases, while the value of MSE is increased when the degree of correlation is increased. However, the MLE has a larger MSE than the PRRE, AUPRRE and MAUPRRE. Table 2 reveals that estimators behave differently with respect to multicollinearity levels, and it is seen that the performance of proposed $\hat{k}_{q4}$ is better than the other estimators. The performance of $\hat{k}_{q1} - \hat{k}_{q3}$ is not superior to the $\hat{k}_{TO}$ when $\rho \le 0.95$ and $q = 3$. In the presence of high but imperfect multicollinearity, the proposed ridge estimators $\hat{k}_{q1} - \hat{k}_{q4}$ are superior to the MLE and $\hat{k}_{TO}$. From Table 3, when $q = 6$ and $\rho > 0.80$, the MAUPRRE with the ridge estimators $\hat{k}_{q1} - \hat{k}_{q4}$ exhibit very good performances since it has lowest MSE values. When the multicollinearity level increases the MSE values of all the estimators increases. But the severe multicollinearity level $\left( {\rho = 0.99} \right)$ does not show a substantial effect on the performance of MAUPRRE with $\hat{k}_{q4}$ as showing for other estimators. The effect of increasing the number of explanatory variables for a given $\rho$ and $n$ leads to an increase in the MSE. When $q = 6$ and $n = 25$, the performance of MLE is very poor. The performance of MAUPPRE $\left( {\hat{k}_{q4} } \right)$ is superior to the MLE, AUPRRE and $\hat{k}_{TO}$ ($\hat{k}_{TO}$ suggested by Türkan and Özel 2016). The simulated absolute bias values of the PPRE, AUPRRE and MAUPRRE are given in Table 4 and 5. $\hat{k}_{q4}$ give minimum bias as compared to other estimators. However, the performance of MAUPRRE is satisfactory in the sense of having the smallest bias (almost unbiased) when we use $\hat{k}_{q4}$ in MAUPRRE. As the sample size and the number of explanatory variables increases the absolute bias of the estimators is decreased. However, the multicollinearity level has a negative effect on the performance of the estimators. Overall, as expected, we can see that the estimated MSE and bias of the estimators increase due to the increase in the multicollinearity level, but the effects of multicollinearity are least problematic when using our new MAUPPRE $\left( {\hat{k}_{q4} } \right)$. The AUPPRE $\left( {\hat{k}_{q4} } \right)$ provides minimum bias when sample size small and $q = 3$. As $q = 6$, $n \to \infty$ and $\rho \to 0.99$, the performance of MAUPPRE $\left( {\hat{k}_{q4} } \right)$ is superior to other estimators in the sense of absolute bias. Finally, when looking at the simulation results, the greatest benefit of applying MAUPRRE is in the situation when ridge estimator $\hat{k}_{q4}$ is used.

**Table 2:** Estimated simulated MSE values when $q = 3$

$\rho$	*n*	MLE	$\hat{k}_{TO}$			$\hat{k}_{q1}$			$\hat{k}_{q2}$			$\hat{k}_{q3}$			$\hat{k}_{q4}$		
			PRRE	AUPRRE	MAUPRRE	PRRE	AUPRRE	MAUPRRE	PRRE	AUPRRE	MAUPRRE	PRRE	AUPRRE	MAUPRRE	PRRE	AUPRRE	MAUPRRE
0.80	25	1.050	0.981	0.994	0.982	0.983	0.979	0.989	0.979	0.981	0.985	0.986	0.977	0.979	0.981	0.980	0.976
	50	0.959	0.845	0.778	0.819	0.899	0.854	0.933	0.855	0.803	0.892	0.902	0.807	0.862	0.834	0.804	0.753
	100	0.941	0.761	0.662	0.729	0.860	0.794	0.904	0.787	0.705	0.834	0.856	0.721	0.804	0.758	0.722	0.643
	200	0.930	0.749	0.645	0.713	0.858	0.791	0.906	0.759	0.678	0.808	0.794	0.657	0.744	0.610	0.607	0.577
	400	0.889	0.599	0.509	0.575	0.758	0.672	0.813	0.631	0.538	0.676	0.650	0.529	0.612	0.506	0.506	0.488
0.90	25	1.272	1.070	1.126	1.051	1.022	1.042	1.014	1.074	1.023	1.037	1.029	1.056	1.018	1.083	1.042	1.026
	50	0.981	0.917	0.924	0.915	0.945	0.931	0.960	0.928	0.908	0.917	0.923	0.908	0.938	0.902	0.898	0.894
	100	0.962	0.869	0.823	0.852	0.916	0.882	0.940	0.902	0.836	0.877	0.885	0.842	0.912	0.797	0.855	0.837
	200	0.929	0.811	0.787	0.803	0.868	0.830	0.891	0.840	0.783	0.821	0.812	0.778	0.828	0.774	0.764	0.755
	400	0.895	0.765	0.709	0.747	0.857	0.801	0.895	0.803	0.718	0.773	0.750	0.700	0.773	0.702	0.688	0.680
0.95	25	1.499	1.246	1.149	1.116	1.056	1.096	1.040	1.146	1.084	1.060	1.071	1.122	1.049	1.161	1.094	1.069
	50	1.199	1.050	0.996	0.980	0.968	0.981	0.967	0.967	0.990	0.961	0.965	0.983	0.961	0.994	0.963	0.954
	100	1.028	0.920	0.916	0.917	0.935	0.920	0.945	0.928	0.918	0.903	0.931	0.921	0.905	0.917	0.890	0.907
	200	0.978	0.934	0.896	0.883	0.901	0.895	0.906	0.875	0.886	0.869	0.876	0.886	0.872	0.901	0.879	0.864
	400	0.943	0.835	0.816	0.806	0.865	0.837	0.882	0.809	0.810	0.804	0.811	0.810	0.806	0.855	0.820	0.810
0.99	25	2.804	1.772	1.440	1.328	1.230	1.147	1.117	1.279	1.184	1.152	1.175	1.276	1.138	1.337	1.212	1.169
	50	2.187	1.487	1.243	1.161	1.120	1.052	1.030	1.139	1.067	1.043	1.145	1.065	1.038	1.180	1.085	1.052
	100	1.565	1.294	1.135	1.082	1.046	1.002	0.988	1.068	1.014	0.998	1.073	1.014	0.995	1.113	1.040	1.007
	200	1.666	1.190	1.073	1.033	1.011	0.989	0.982	1.024	0.990	0.983	1.020	0.992	0.983	1.057	1.007	0.946
	400	1.267	1.094	1.028	1.009	0.980	0.955	0.947	1.011	0.975	0.961	1.015	0.967	0.952	1.033	0.997	0.884

**Table 3:** Estimated simulated MSE values when $q = 6$

$\rho$	*n*	MLE	$\hat{k}_{TO}$			$\hat{k}_{q1}$			$\hat{k}_{q2}$			$\hat{k}_{q3}$			$\hat{k}_{q4}$		
			PRRE	AUPRRE	MAUPRRE	PRRE	AUPRRE	MAUPRRE	PRRE	AUPRRE	MAUPRRE	PRRE	AUPRRE	MAUPRRE	PRRE	AUPRRE	MAUPRRE
0.80	25	2.014	1.170	1.377	1.112	1.017	1.070	1.007	1.079	1.222	1.041	1.044	1.151	1.018	1.314	1.121	1.066
	50	1.145	0.914	0.949	0.905	0.945	0.958	0.929	0.907	0.914	0.909	0.918	0.909	0.901	0.937	0.898	0.882
	100	0.929	0.825	0.850	0.811	0.886	0.905	0.855	0.820	0.819	0.813	0.855	0.830	0.808	0.847	0.812	0.790
	200	0.924	0.795	0.778	0.801	0.897	0.862	0.853	0.816	0.834	0.775	0.826	0.825	0.779	0.782	0.770	0.756
	400	0.890	0.759	0.780	0.746	0.863	0.827	0.816	0.761	0.743	0.754	0.763	0.762	0.740	0.772	0.751	0.732
0.90	25	3.193	1.461	1.882	1.329	1.089	1.185	1.063	1.257	1.522	1.178	1.361	1.174	1.121	1.630	1.311	1.214
	50	1.474	1.011	1.099	0.983	0.971	0.981	0.971	0.974	1.022	0.958	0.997	0.965	0.955	1.071	0.989	0.961
	100	1.104	0.944	1.005	0.921	0.929	0.925	0.901	0.913	0.956	0.892	0.929	0.904	0.889	0.995	0.930	0.905
	200	1.126	0.893	0.944	0.875	0.907	0.883	0.871	0.914	0.871	0.865	0.889	0.861	0.849	0.944	0.887	0.866
	400	1.006	0.862	0.890	0.849	0.892	0.882	0.825	0.855	0.849	0.840	0.865	0.842	0.832	0.882	0.866	0.827
0.95	25	5.383	2.589	1.803	1.538	1.169	1.317	1.126	1.400	1.790	1.275	1.577	1.300	1.217	2.029	1.509	1.335
	50	1.973	1.252	1.102	1.053	1.000	1.030	0.991	1.116	1.033	1.006	1.076	1.020	0.994	1.177	1.061	1.024
	100	1.448	1.113	1.034	1.009	0.968	0.984	0.961	1.053	0.990	0.966	1.014	0.977	0.951	1.097	1.018	0.993
	200	1.331	1.034	0.974	0.956	0.933	0.944	0.927	0.999	0.943	0.921	0.972	0.929	0.907	1.036	0.970	0.951
	400	1.128	1.013	0.928	0.896	0.915	0.906	0.919	0.915	0.889	0.876	0.899	0.887	0.879	0.961	0.900	0.874
0.99	25	22.424	7.684	3.943	2.587	1.664	1.363	1.269	2.966	1.856	1.450	2.417	1.655	1.387	4.373	2.376	1.622
	50	5.484	1.936	1.416	1.228	1.147	1.073	1.051	1.350	1.169	1.110	1.258	1.128	1.087	1.520	1.238	1.139
	100	3.814	1.656	1.221	1.150	1.102	1.052	1.036	1.206	1.121	1.096	1.174	1.099	1.077	1.276	1.157	1.125
	200	2.389	1.364	1.204	1.081	1.045	1.009	0.997	1.122	1.059	1.042	1.104	1.043	1.024	1.261	1.099	1.085
	400	1.534	1.165	1.098	1.060	0.984	0.951	0.939	1.074	0.976	0.940	1.047	0.965	0.935	1.161	1.049	0.943

**Table 4:** Estimated absolute Bias values when $q = 3$

$\rho$	*n*	$\hat{k}_{TO}$			$\hat{k}_{q1}$			$\hat{k}_{q2}$			$\hat{k}_{q3}$			$\hat{k}_{q4}$		
		PRRE	AUPRRE	MAUPRRE	PRRE	AUPRRE	MAUPRRE	PRRE	AUPRRE	MAUPRRE	PRRE	AUPRRE	MAUPRRE	PRRE	AUPRRE	MAUPRRE
0.80	25	0.331	0.327	0.327	0.326	0.328	0.330	0.327	0.328	0.326	0.326	0.326	0.329	0.254	0.127	0.151
	50	0.282	0.259	0.273	0.285	0.300	0.311	0.268	0.297	0.285	0.269	0.287	0.301	0.207	0.109	0.115
	100	0.254	0.221	0.243	0.265	0.287	0.301	0.235	0.278	0.262	0.240	0.268	0.285	0.141	0.064	0.105
	200	0.250	0.215	0.238	0.264	0.286	0.302	0.226	0.269	0.253	0.219	0.248	0.265	0.102	0.023	0.063
	400	0.200	0.170	0.192	0.224	0.253	0.271	0.179	0.225	0.210	0.176	0.204	0.217	0.088	0.006	0.018
0.90	25	0.375	0.357	0.350	0.347	0.341	0.338	0.358	0.341	0.346	0.352	0.343	0.339	0.261	0.147	0.191
	50	0.308	0.305	0.306	0.310	0.315	0.320	0.303	0.309	0.306	0.303	0.308	0.313	0.219	0.120	0.160
	100	0.274	0.284	0.290	0.294	0.305	0.313	0.279	0.301	0.292	0.281	0.295	0.304	0.179	0.107	0.129
	200	0.262	0.268	0.270	0.277	0.289	0.297	0.261	0.280	0.274	0.259	0.271	0.276	0.128	0.095	0.107
	400	0.236	0.249	0.255	0.267	0.286	0.298	0.239	0.268	0.258	0.233	0.250	0.258	0.103	0.089	0.066
0.95	25	0.415	0.383	0.372	0.365	0.352	0.347	0.382	0.353	0.361	0.374	0.357	0.350	0.287	0.165	0.216
	50	0.350	0.332	0.327	0.327	0.323	0.322	0.330	0.320	0.322	0.328	0.322	0.320	0.231	0.151	0.132
	100	0.311	0.305	0.306	0.307	0.312	0.315	0.301	0.309	0.306	0.302	0.307	0.310	0.206	0.113	0.102
	200	0.307	0.299	0.294	0.298	0.300	0.302	0.295	0.290	0.292	0.295	0.292	0.291	0.166	0.103	0.088
	400	0.278	0.272	0.269	0.279	0.288	0.294	0.270	0.268	0.270	0.270	0.270	0.269	0.148	0.097	0.007
0.99	25	0.591	0.480	0.443	0.410	0.382	0.372	0.426	0.384	0.395	0.425	0.392	0.379	0.346	0.190	0.180
	50	0.496	0.414	0.387	0.373	0.351	0.343	0.380	0.348	0.356	0.382	0.355	0.346	0.334	0.162	0.151
	100	0.431	0.378	0.361	0.349	0.334	0.329	0.356	0.333	0.338	0.358	0.338	0.332	0.271	0.147	0.134
	200	0.397	0.358	0.344	0.337	0.330	0.327	0.341	0.328	0.330	0.340	0.331	0.328	0.182	0.116	0.063
	400	0.365	0.343	0.336	0.327	0.318	0.316	0.337	0.320	0.325	0.338	0.322	0.317	0.153	0.100	0.008

**Table 5:** Estimated absolute Bias values when $q = 6$

$\rho$	*n*	$\hat{k}_{TO}$			$\hat{k}_{q1}$			$\hat{k}_{q2}$			$\hat{k}_{q3}$			$\hat{k}_{q4}$		
		PRRE	AUPRRE	MAUPRRE	PRRE	AUPRRE	MAUPRRE	PRRE	AUPRRE	MAUPRRE	PRRE	AUPRRE	MAUPRRE	PRRE	AUPRRE	MAUPRRE
0.8	25	0.275	0.234	0.222	0.203	0.214	0.201	0.216	0.208	0.244	0.230	0.209	0.204	0.190	0.108	0.114
	50	0.190	0.183	0.181	0.189	0.186	0.192	0.181	0.182	0.183	0.180	0.182	0.184	0.168	0.087	0.099
	100	0.170	0.165	0.162	0.179	0.171	0.185	0.164	0.167	0.164	0.162	0.166	0.171	0.148	0.059	0.046
	200	0.156	0.159	0.160	0.177	0.171	0.181	0.163	0.163	0.155	0.156	0.165	0.165	0.129	0.015	0.001
	400	0.156	0.152	0.149	0.173	0.163	0.178	0.151	0.149	0.152	0.148	0.153	0.152	0.127	0.006	0.000
0.9	25	0.376	0.292	0.266	0.218	0.237	0.213	0.251	0.236	0.304	0.272	0.224	0.235	0.202	0.113	0.123
	50	0.220	0.202	0.197	0.194	0.196	0.194	0.195	0.192	0.204	0.199	0.191	0.193	0.180	0.094	0.108
	100	0.201	0.189	0.184	0.186	0.185	0.186	0.183	0.178	0.191	0.186	0.178	0.181	0.152	0.073	0.058
	200	0.189	0.179	0.175	0.181	0.177	0.184	0.174	0.171	0.183	0.178	0.172	0.173	0.134	0.020	0.015
	400	0.178	0.172	0.170	0.178	0.176	0.179	0.171	0.170	0.170	0.172	0.168	0.168	0.110	0.009	0.005
0.95	25	0.518	0.361	0.308	0.234	0.263	0.225	0.280	0.255	0.358	0.315	0.260	0.243	0.220	0.141	0.124
	50	0.250	0.220	0.211	0.200	0.206	0.198	0.207	0.201	0.223	0.215	0.203	0.199	0.181	0.124	0.092
	100	0.223	0.207	0.202	0.194	0.197	0.192	0.198	0.193	0.211	0.204	0.194	0.190	0.180	0.119	0.075
	200	0.207	0.195	0.191	0.187	0.189	0.185	0.189	0.184	0.200	0.195	0.186	0.181	0.144	0.089	0.041
	400	0.203	0.186	0.179	0.183	0.181	0.184	0.178	0.175	0.183	0.180	0.177	0.176	0.109	0.021	0.007
0.99	25	1.537	0.789	0.517	0.333	0.273	0.254	0.371	0.290	0.593	0.483	0.277	0.331	0.255	0.175	0.167
	50	0.387	0.283	0.246	0.229	0.215	0.210	0.234	0.222	0.270	0.252	0.217	0.226	0.208	0.130	0.105
	100	0.331	0.244	0.230	0.220	0.210	0.207	0.224	0.219	0.241	0.235	0.215	0.220	0.180	0.103	0.075
	200	0.273	0.241	0.216	0.209	0.202	0.199	0.212	0.208	0.224	0.221	0.205	0.209	0.153	0.085	0.052
	400	0.233	0.220	0.212	0.197	0.190	0.188	0.195	0.188	0.215	0.209	0.187	0.193	0.120	0.023	0.009

## Application: Swedish Football League 2019

**Fig. 1:**
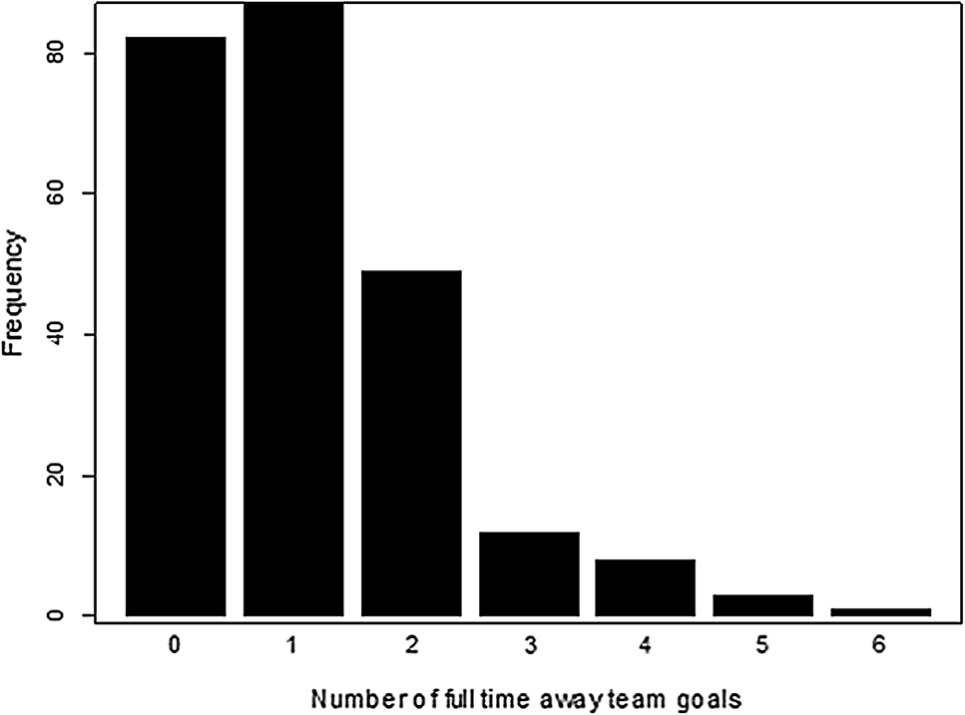
Distribution of number of away-team goals (within full time)

For the purpose of illustrating the empirical relevance of the proposed methods, we analyze Swedish football data.^1^ The proposed and existing estimation methods are explicated using a dataset regarding the performance of Swedish football teams in the top Swedish league (Allsvenskan) during the year of 2019. This dataset includes $n = 242$ observations which include one dependent and six explanatory variables. These variables are defined as: number of, within full time, away-team goals (*y*), pinnacle home win odds $\left( {x_{1} } \right)$, pinnacle draw odds $\left( {x_{2} } \right)$, pinnacle away win odds $\left( {x_{3} } \right)$, maximum market home win odds $\left( {x_{4} } \right)$, maximum market draw win odds $\left( {x_{4} } \right)$ and maximum market away win odds $\left( {x_{6} } \right)$. The effects of the regressors $\left( {x_{1} \;{\text{to}}\; x_{6} } \right)$ on the dependent variable are analyzed using the PRM. The distribution of the dependent variable is illustrated in Fig. 1 which indicates that the PRM is well fitted. Based on a Chi-square $\left( {\chi^{2} } \right)$ goodness of fit test, the results confirm that the response variable is well suited to the PRM (with a *p* value = 0.15). The correlation matrix of the regressors is exhibited in Table 6. Table 6 shows severe correlation among $x_{1}$, $x_{3}$, $x_{4}$ and $x_{6}$. Furthermore, the condition number, which is the ratio of maximum to the minimum eigenvalues, is $1766 > 1000$ which indicates what can be defined as a severe multicollinearity problem in this dataset.

**Table 6:** Correlation Matrix

Variables	$x_{1}$	$x_{2}$	$x_{3}$	$x_{4}$	$x_{5}$	$x_{6}$
$x_{1}$	1.000					
$x_{2}$	0.077	1.000				
$x_{3}$	− 0.563	0.708	1.000			
$x_{4}$	0.997	0.098	− 0.548	1.000		
$x_{5}$	0.034	0.988	0.755	0.054	1.000	
$x_{6}$	− 0.539	0.738	0.990	− 0.524	0.783	1.000

**Fig. 2:**
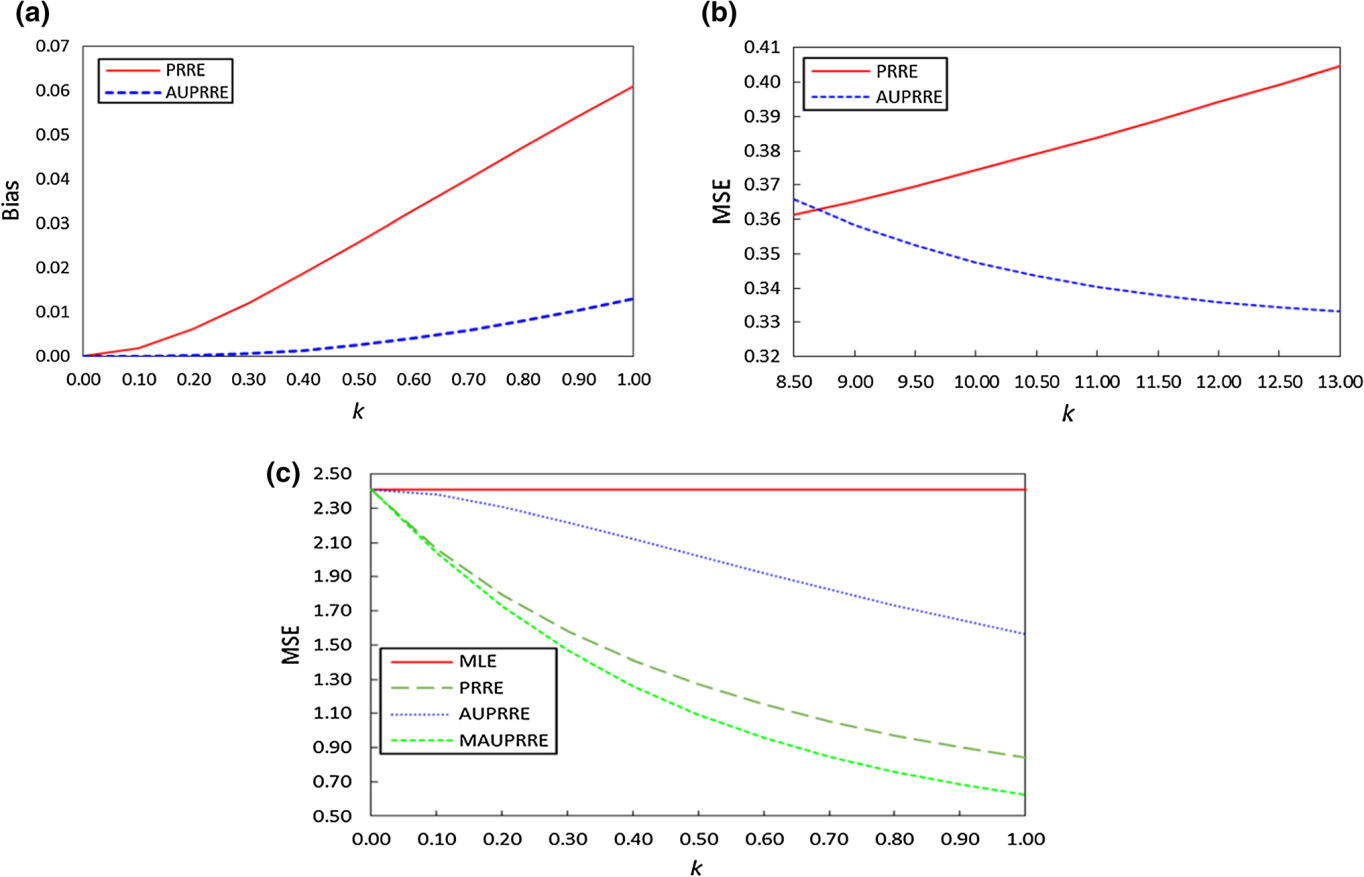
**a** Squared bias values of PRRE and AUPRRE versus *k*; **b** MSE values of MLE and AUPRRE versus *k*; **c** MSE values of MLE, PRRE, AUPRRE and MAUPRRE versus *k*

We present the coefficients and the standard errors of the estimators in Table 7. The MSE and bias values of the estimators are illustrated in Fig. 2a–c. Theoretical MSE values of the $\tilde{\beta }_{\text{MLE}}$, $\tilde{\beta }_{\text{PRRE}}$, $\tilde{\beta }_{\text{AUPRRE}}$ and $\tilde{\beta }_{\text{MAUPRRE}}$ are calculated using Eqs. (6), (11), (16) and (21), respectively. Simulation results revealed that the performance of the ridge estimator $\hat{k}_{q4}$ is an efficient and $\hat{k}_{q4}$ exhibited minimum MSE compared to other estimators. Therefore, we use $\hat{k}_{q4}$ in the $\tilde{\beta }_{\text{PRRE}}$, $\tilde{\beta }_{\text{AUPRRE}}$ and $\tilde{\beta }_{\text{MAUPRRE}}$ for estimation of the PRM. For comparison purposes, we also use $\hat{k}_{TO}$ from Türkan and Özel (2016). The effects of the estimated coefficients are changed, and the estimated standard errors of the $\tilde{\beta }_{\text{MAUPRRE}}$ are smaller than those of $\tilde{\beta }_{\text{AUPRRE}}$, $\tilde{\beta }_{\text{PRRE}}$, $\tilde{\beta }_{\text{MLE}}$. It is evident from Table 7, based on high standard errors, that the MLE does not estimate the coefficients very precisely in the presence of multicollinearity. However, on the other hand, the proposed estimation method $\tilde{\beta }_{\text{MAUPRRE}} \left( {\hat{k}_{q4} } \right)$ estimates the coefficients rather precisely. The PRRE provides smaller standard errors as compared to the AUPRRE and MLE. The AUPRRE gives higher standard errors of the parameters since AUPRRE provides minimum squared bias and MSE among other estimators under certain conditions. AUPRRE shrinks the bias, and therefore, we named it almost unbiased estimator due to its minimized bias. One can easily see that in the presence of multicollinearity MLE exhibits the wrong sign of the slope parameters $\tilde{\beta }_{3}$ and $\tilde{\beta }_{6}$. However, biased estimation methods may change the sign of the slope parameters. For instance, theoretically, pinnacle away win odds and maximum market away win odds have negative effects on the number of fulltime away-team goals, while the MLE shows a negative effect. Meanwhile, proposed method shows positive effect and it is considered a good approach to tackle the problem of multicollinearity. Hence, the advantage of the proposed method over MLE using this empirical application is easily understood.

**Table 7:** Estimated regression coefficients and standard errors of the estimators

Estimators	$\tilde{\beta }_{MLE}$	$\hat{k}_{TO}$			$\hat{k}_{q4}$		
		$\tilde{\beta }_{PRRE}$	$\tilde{\beta }_{AUPRRE}$	$\tilde{\beta }_{MAUPRRE}$	$\tilde{\beta }_{PRRE}$	$\tilde{\beta }_{AUPRRE}$	$\tilde{\beta }_{MAUPRRE}$
*Coefficient estimates*							
$\tilde{\beta }_{o}$	0.020	− 0.180	− 0.180	− 0.179	− 0.178	− 0.180	− 0.178
$\tilde{\beta }_{1}$	0.522	0.150	0.157	0.149	0.146	0.151	0.146
$\tilde{\beta }_{2}$	− 0.553	− 0.037	− 0.038	− 0.036	− 0.034	− 0.037	− 0.034
$\tilde{\beta }_{3}$	− 0.175	0.229	0.290	0.212	0.156	0.233	0.117
$\tilde{\beta }_{4}$	− 0.531	− 0.205	− 0.370	− 0.072	− 0.084	− 0.162	− 0.013
$\tilde{\beta }_{5}$	0.899	0.088	0.162	0.026	0.035	0.069	0.004
$\tilde{\beta }_{6}$	− 0.539	0.079	0.149	0.017	0.031	0.060	0.002
*Standard errors*							
$se\left( {\tilde{\beta }_{o} } \right)$	0.067	0.024	0.024	0.024	0.024	0.024	0.024
$se\left( {\tilde{\beta }_{1} } \right)$	0.615	0.041	0.042	0.041	0.040	0.040	0.040
$se\left( {\tilde{\beta }_{2} } \right)$	0.495	0.067	0.069	0.067	0.063	0.069	0.063
$se\left( {\tilde{\beta }_{3} } \right)$	0.688	0.165	0.209	0.153	0.112	0.169	0.084
$se\left( {\tilde{\beta }_{4} } \right)$	0.626	0.149	0.268	0.052	0.061	0.118	0.009
$se\left( {\tilde{\beta }_{5} } \right)$	0.592	0.138	0.254	0.041	0.055	0.108	0.007
$se\left( {\tilde{\beta }_{6} } \right)$	0.750	0.121	0.227	0.026	0.047	0.092	0.004

We also plot the squared bias and MSE values using Eqs. (11), (16) and (21) against assuming different values of *k* to show the performance of estimators under different conditions. In Fig. 2a, we plot the squared bias values of the PRRE and AUPRRE for changing the values of the ridge parameter *k* between 0 and 1. It is seen that AUPRRE has always the minimum bias compared to the PRRE, and these results satisfy Theorem 3.1.1 when the values of $k > 0$. The estimated MSE values of the PRRE and AUPRRE are shown in Fig. 2b. The AUPRRE should have less MSE than the PRRE when $k > 8.748$ and these findings satisfy Theorem 3.1.2. However, we also plot the MSE values of the MLE, PRRE, AUPRRE and MAUPRRE to exemplify Theorems 3.2.1–3.2.3 in Fig. 2c. It is found that the MSE of the biased estimators equals to MLE when $k = 0$. As the value of *k* increases, the MSE of MAUPRRE demonstrate the minimum MSE compared to the AUPRRE, PRRE and MLE. Therefore, we can conclude that the performance of the PRRE, AUPRRE and MAUPRRE is a function of the values of the ridge estimators. Overall, we recommend practitioners to apply MAUPRRE with ridge estimator $\hat{k}_{q4}$ since this estimator gives lowest standard errors and MSE in the presence of multicollinearity.

## Conclusions

In this paper, we derive the MSE properties of the AUPRRE and MAUPRRE to show the superiority over the existing estimators in the presence of multicollinearity. We also derive the optimal ridge parameter, *k* by minimizing the MSE of AUPRRE and suggest new ridge estimators. These estimators are based on the proposed optimal value of *k* for estimating of the ridge parameter, *k*, which we demonstrate to exhibit superiority over the existing estimators. The comparison of the proposed estimators is made using the AUPRRE, PRRE and MLE by means of Monte Carlo simulations. The comparison concludes that MAUPRRE with the ridge estimator $\hat{k}_{q4}$ has a smaller MSE than MLE, PRRE and AUPRRE. Moreover, the empirical relevance and appealing properties of the proposed estimator are also demonstrated by utilizing our approach on a collinear real-world application. In conclusion, both empirically and by using simulations, in the presence of multicollinearity our MAUPRRE ($\hat{k}_{q4}$) approach exhibits the lowest MSE compared to all competing estimators.

## Electronic Supplementary Material

Below is the link to the electronic supplementary material.

Supplementary material 1 (PDF 112 kb)

## Footnotes

1 The dataset is taken from Sweden football results. The homepage is www.football-data.co.uk.
