# Supplementary material for: Biased Adjusted Poisson Ridge Estimators-Method and Application
Source: Iran J Sci Technol Trans A Sci. 2020 Oct 3;44(6):1775–89. doi: 10.1007/s40995-020-00974-5 (PMC7532743; doi:10.1007/s40995-020-00974-5)
Supplement: Supplementary file 1 — Supplementary material 1 (PDF 112 kb) [file 40995_2020_974_MOESM1_ESM.pdf]

Appendix: R code of the real data

da= read.table(file.choose())	#import data
dataset<-attach(da)	#defines the dataset
q=6	#defines the number of explanatory variables in the model (in this case 6 variables)
P=q+1	
n=242	#sample size
X = cbind(V2,V3,V4,V5,V6,V7)	#defines a matrix containing all the regressors
X=scale(X,center = TRUE,scale = TRUE)	#scaled matrix
model = glm(V1~X,family=poisson(link="log"))	#estimates the Poisson regression model using Maximum Likelihood
c=coefficients(model)	#extracts the estimates of the coefficients estimated by Maximum Likelihood
w = model$weight	#extracts the weight matrix
W = diag(w)	
yhat=model$fit	#extracts the fitted value from the model
x=cbind(1,X)	#defines a matrix containing constant term
X1 = cbind(x)	
xw = t(X1)%*%W%*%X1	#the estimated variance-covariance matrix estimated by Maximum Likelihood
Q=eigen(xw)$vectors	#calculates eigenvectors from $X^t\widehat{W}X$
M=X1%*%Q	
l=(t(M)%*%W%*%M)	#calculates $\Lambda = \mathrm{diag}(\lambda_1, \lambda_2, \dots, \lambda_q) = \mathcal{Z}^t\widehat{W}\mathcal{Z}$
L=diag(eigen(l)$values)	
u=log(yhat)+((V1-yhat)/(yhat))	#estimates the adjusted response variable
ml=cbind(solve(L)%*%t(M)%*%W%*%u)	#estimates the $\tilde{\gamma}_{MLE}$
ML=Q%*%ml	#calculates the estimated parameters by Maximum Likelihood
lemda =(eigen(l)$values)	#extractes the eigenvalues from $X^t\widehat{W}X$
alpha = Q%*%ML	#calcualtes alfa parameters
asq=alpha^2	#taking the square of the vector containing the alfa parameters
sig=(sum((V1-yhat)^2))/(n-P)	#calcualtes $\hat{\sigma}^2$
mi=sqrt(sig/asq)	#calculates shrinkage parameter $\hat{k}_{TO}$
k=median(mi^2)	
I=diag(P)	#creates an identity matrix of dimension (P)*(P)
B=solve(L+ k*I)	#calculates $\left(\Lambda_{kI_q}\right)^{-1}$
PRRE=(I-k*B)%*%ml	#calculates Poisson ridge regression estimator
AUPRRE=(I-((k*B)^2))%*%ml	#calculates Almost Unbiased Poisson ridge regression estimator
MAUPRRE =(I-((k*B)^2))%*%(I-(k*B))%*%ml	#calculates Modified Almost Unbiased Poisson ridge regression estimator
print(ML)	
print(PRRE)	
print(AUPRRE)	
print(MAUPRRE)	
ki=(sig+sqrt(sig+(asq*lemda)))/(asq)	#calculates shrinkage parameter $\hat{k}_{q4}$
kq4= (prod(ki))^(1/P)	

```
B=solve(L+ kq4*I)                                   #calculates (Λ_{kI_q})^-1
PRRE_kq4=(I- kq4*B)%*%ml                            #calculates Poisson ridge regression estimator
AUPRRE_kq4=(I-((kq4*B)^2))%*%ml                     #calculates Almost Unbiased Poisson ridge regression estimator
MAUPRRE_kq4=(I-((kq4*B)^2))%*%(I-(kq4*B))%*%ml      #calculates Modified Almost Unbiased Poisson ridge regression estimator
print(PRRE_kq4)
print(AUPRRE_kq4)
print(MAUPRRE_kq4)
```
